# Bis[μ-2-(4-hy­droxy­phen­yl)acetato]-κ^3^ *O*,*O*′:*O*;κ^3^ *O*:*O*,*O*′-bis­{aqua­(4,4′-bipyridine-κ*N*)bis­[2-(4-hy­droxy­phen­yl)acetato-κ^2^ *O*,*O*′]ytterbium(III)} monohydrate

**DOI:** 10.1107/S1600536810043643

**Published:** 2010-10-31

**Authors:** Jia-Lu Liu, Jian-Feng Liu, Guo-Liang Zhao

**Affiliations:** a College of Chemistry and Life Sciences, Zhejiang Normal University, Jinhua 321004, People’s Republic of China, and Zhejiang Normal University Xingzhi College, Jinhua 321004, People’s Republic of China

## Abstract

In the title dinuclear complex, [Yb_2_(C_8_H_7_O_3_)_6_(C_10_H_8_N_2_)_2_(H_2_O)_2_]·H_2_O, the Yb^III^ atoms are coordinated by eight O atoms from four 2-(4-hy­droxy­phen­yl)acetate (HPAA) ligands and a water mol­ecule, and one N atom from a 4,4′-bipyridine (bipy) ligand in a distorted tricapped trigonal–prismatic geometry. Whereas four HPAA ligands coordinate to just two Yb^III^ atoms, the remaining two ligands bridge the two Yb^III^ atoms. In the crystal structure, O—H⋯O and O—H⋯N hydrogen bonds link the mol­ecules into a three-dimensional network.

## Related literature

For applications of carb­oxy­lic metal–organic complexes, see: Liu *et al.* (2010 ▶); Wang & Sevov (2008 ▶); Fang & Zhang (2006 ▶); Wang *et al.* (2010 ▶).
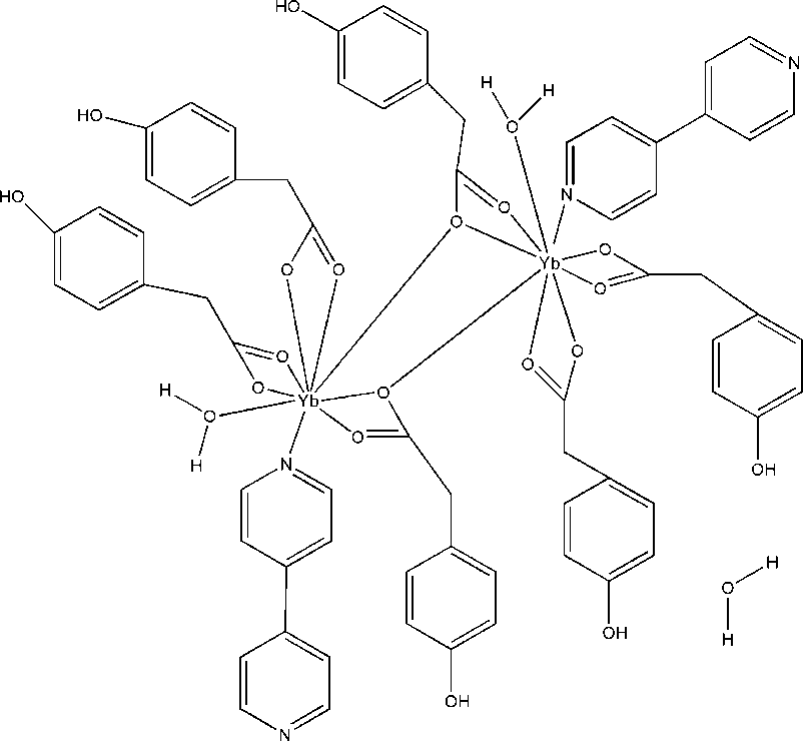


## Experimental

### 

#### Crystal data

[Yb_2_(C_8_H_7_O_3_)_6_(C_10_H_8_N_2_)_2_(H_2_O)_2_]·H_2_O
*M* *_r_* = 1619.31
Triclinic, 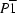

*a* = 11.6885 (1) Å
*b* = 16.1322 (2) Å
*c* = 18.456 (2) Å
α = 83.269 (1)°
β = 72.377 (1)°
γ = 71.441 (1)°
*V* = 3143.4 (3) Å^3^
*Z* = 2
Mo *K*α radiation
μ = 3.04 mm^−1^
*T* = 296 K
0.15 × 0.13 × 0.12 mm

#### Data collection

Bruker APEXII area-detector diffractometer
Absorption correction: multi-scan (*SADABS*; Sheldrick, 1996 ▶) *T*_min_ = 0.640, *T*_max_ = 0.694
41713 measured reflections
11017 independent reflections
8916 reflections with *I* > 2σ(*I*)
*R*_int_ = 0.039

#### Refinement

*R*[*F* ^2^ > 2σ(*F* ^2^)] = 0.025
*wR*(*F* ^2^) = 0.070
*S* = 1.12
11017 reflections
874 parameters
9 restraints
H atoms treated by a mixture of independent and constrained refinement
Δρ_max_ = 0.59 e Å^−3^
Δρ_min_ = −0.66 e Å^−3^

### 

Data collection: *APEX2* (Bruker, 2006 ▶); cell refinement: *SAINT* (Bruker, 2006 ▶); data reduction: *SAINT*; program(s) used to solve structure: *SHELXS97* (Sheldrick, 2008 ▶); program(s) used to refine structure: *SHELXL97* (Sheldrick, 2008 ▶); molecular graphics: *XP* (Sheldrick, 2008 ▶); software used to prepare material for publication: *SHELXL97*.

## Supplementary Material

Crystal structure: contains datablocks I, global. DOI: 10.1107/S1600536810043643/bt5388sup1.cif

Structure factors: contains datablocks I. DOI: 10.1107/S1600536810043643/bt5388Isup2.hkl

Additional supplementary materials:  crystallographic information; 3D view; checkCIF report

## Figures and Tables

**Table 1:** Hydrogen-bond geometry (Å, °)

*D*—H⋯*A*	*D*—H	H⋯*A*	*D*⋯*A*	*D*—H⋯*A*
O6—H6*B*⋯O15^i^	0.82	1.90	2.715 (4)	174
O9—H9*A*⋯O3^ii^	0.82	1.94	2.761 (5)	173
O12—H12*A*⋯O18^iii^	0.82	1.92	2.735 (5)	171
O15—H15*C*⋯O3*W*	0.82	1.85	2.645 (5)	162
O2*W*—H2*WA*⋯N2^ii^	0.85 (2)	1.98 (2)	2.820 (5)	176 (6)
O1*W*—H1*WA*⋯O4	0.83 (5)	1.95 (3)	2.731 (4)	156 (5)
O2*W*—H2*WB*⋯O13	0.84 (6)	2.02 (3)	2.741 (4)	144 (5)
O1*W*—H1*WB*⋯N4^iii^	0.84 (2)	1.98 (3)	2.779 (5)	159 (6)
O3*W*—H3*WA*⋯O12^ii^	0.81 (6)	2.01 (3)	2.797 (5)	164 (7)

Symmetry codes: (i) 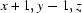; (ii) 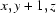; (iii) 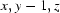.

**Fig. 1.**
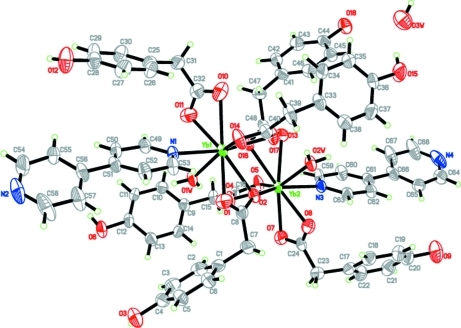
The molecular structure of the title complex, showing the atom-labelling scheme. Displacement ellipsoids are drawn at the 50% probability level.

## supplementary crystallographic information

### Comment

The design and synthesis of carboxylic metal–organic complexes have been of an increasing interest for decades owing to their potential practical applications in fluorescence or magnetism (Wang *et al.*, 2010; Fang & Zhang, 2006; Wang & Sevov, 2008; Liu *et al.*, 2010). The title compound consists of six HPPA ligands, two bipy molecules and three water molecules. In the bicentric complex, every Yb atom is coordinated by seven O atoms from four HPPA ligands, one N atom from 4,4'-bipyridine ligand and one O atom from a water molecule. The Yb atom is nine coordinated.  The Yb^III^ atom is in a distorted capped pentagonal prism environment. The Yb—O bond lengths range from 2.297 (3)–2.569 (3) Å. The Yb—N distances range from 2.494 (3)–2.509 (3) Å. The Yb—O(water) bond lengths range from 2.327 (3)–2.338 (3) Å, is slightly shorter than other Yb—O bonds. In addition, there are plenty of hydrogen bonds in the crystal structure due to the existence of dissociative water and crystal water molecules. The occurrence of numerous O—H···O involving coordinated and non-coordinated water molecules build up an intricated three-dimensional network.

### Experimental

All reagents and solvents used were of commercially available quality and without purified before using. *p*-hydroxyphenylacetic acid (HPAA) (0.456 g, 3 mmol) and sodium hydroxide (0.12 g, 3 mmol) were mixed together in water (10 ml), then Yb[(NO_3_)_3_] (0.360 g, 1 mmol) dissolved in water (10 ml) was added to the above solution. After stirring for one hour, an ethanol (5 ml) solution of 4,4'-bipyridine (0.156 g, 1 mmol) was slowly dripped into the above solution with stirring for three hours. After filtration, the filtrate was allowed to stand at room temperature, and single crystals suitable were obtained after one week.

### Refinement

H atoms excluding the water H atoms were geometrically positioned and treated as riding with C—H = 0.97 Å (methylene) or 0.93 Å (aromatic) and O—H = 0.82 Å with *U*_iso_(H) = 1.2*U*_eq_(C) or *U*_iso_(H) = 1.5*U*_eq_(O). The H atoms of the water molecules were located in a difference Fourier map. Their coordinates were refined using restraints [O—H = 0.82 (1)Å and H···H = 1.39 (2) Å] with *U*_iso_(H) = 1.5*U*_eq_(O).

### Crystal data

**Table:**

[Yb_2_(C_8_H_7_O_3_)_6_(C_10_H_8_N_2_)_2_(H_2_O)_2_]·H_2_O	*Z* = 2
*M**_r_* = 1619.31	*F*(000) = 1616
Triclinic, *P*1	*D*_x_ = 1.711 Mg m^−^^3^
Hall symbol: -P 1	Mo *K*α radiation, λ = 0.71073 Å
*a* = 11.6885 (1) Å	Cell parameters from 9903 reflections
*b* = 16.1322 (2) Å	θ = 1.2–25.0°
*c* = 18.456 (2) Å	µ = 3.04 mm^−^^1^
α = 83.269 (1)°	*T* = 296 K
β = 72.377 (1)°	Block, colourless
γ = 71.441 (1)°	0.15 × 0.13 × 0.12 mm
*V* = 3143.4 (3) Å^3^	

### Data collection

**Table:**

Bruker APEXII area-detector diffractometer	11017 independent reflections
Radiation source: fine-focus sealed tube	8916 reflections with *I* > 2σ(*I*)
graphite	*R*_int_ = 0.039
φ and ω scans	θ_max_ = 25.0°, θ_min_ = 1.2°
Absorption correction: multi-scan (*SADABS*; Sheldrick, 1996)	*h* = −13→13
*T*_min_ = 0.640, *T*_max_ = 0.694	*k* = −19→17
41713 measured reflections	*l* = −21→21

### Refinement

**Table:**

Refinement on *F*^2^	Primary atom site location: structure-invariant direct methods
Least-squares matrix: full	Secondary atom site location: difference Fourier map
*R*[*F*^2^ > 2σ(*F*^2^)] = 0.025	Hydrogen site location: inferred from neighbouring sites
*wR*(*F*^2^) = 0.070	H atoms treated by a mixture of independent and constrained refinement
*S* = 1.12	*w* = 1/[σ^2^(*F*_o_^2^) + (0.0324*P*)^2^ + 0.1358*P*] where *P* = (*F*_o_^2^ + 2*F*_c_^2^)/3
11017 reflections	(Δ/σ)_max_ = 0.002
874 parameters	Δρ_max_ = 0.59 e Å^−^^3^
9 restraints	Δρ_min_ = −0.66 e Å^−^^3^

### Special details

**Table:**

Geometry. All e.s.d.'s (except the e.s.d. in the dihedral angle between two l.s. planes) are estimated using the full covariance matrix. The cell e.s.d.'s are taken into account individually in the estimation of e.s.d.'s in distances, angles and torsion angles; correlations between e.s.d.'s in cell parameters are only used when they are defined by crystal symmetry. An approximate (isotropic) treatment of cell e.s.d.'s is used for estimating e.s.d.'s involving l.s. planes.
Refinement. Refinement of *F*^2^ against ALL reflections. The weighted *R*-factor *wR* and goodness of fit *S* are based on *F*^2^, conventional *R*-factors *R* are based on *F*, with *F* set to zero for negative *F*^2^. The threshold expression of *F*^2^ > σ(*F*^2^) is used only for calculating *R*-factors(gt) *etc*. and is not relevant to the choice of reflections for refinement. *R*-factors based on *F*^2^ are statistically about twice as large as those based on *F*, and *R*-factors based on ALL data will be even larger.

### Fractional atomic coordinates and isotropic or equivalent isotropic displacement parameters (Å^2^)

**Table:**

	*x*	*y*	*z*	*U*_iso_*/*U*_eq_	
Yb1	0.227023 (15)	0.136366 (10)	0.219116 (10)	0.02695 (6)	
Yb2	0.369022 (15)	0.291979 (10)	0.301700 (9)	0.02563 (6)	
N1	0.1895 (3)	−0.0073 (2)	0.21880 (19)	0.0312 (8)	
N2	0.1122 (4)	−0.4310 (3)	0.2713 (3)	0.0636 (13)	
N3	0.4109 (3)	0.4338 (2)	0.30794 (18)	0.0316 (8)	
N4	0.4624 (5)	0.8643 (3)	0.2729 (3)	0.0650 (14)	
O1W	0.4082 (3)	0.04386 (17)	0.24614 (18)	0.0357 (7)	
O1	0.1484 (3)	0.1018 (2)	0.34968 (16)	0.0454 (8)	
O2W	0.1795 (3)	0.38500 (18)	0.28430 (18)	0.0384 (7)	
O2	0.2446 (2)	0.20067 (16)	0.33258 (15)	0.0310 (6)	
O3	0.3306 (3)	−0.21343 (19)	0.56157 (19)	0.0557 (9)	
H3B	0.4017	−0.2278	0.5668	0.084*	
O3W	−0.0538 (4)	0.7316 (3)	0.0107 (2)	0.0675 (10)	
O4	0.5119 (2)	0.14943 (16)	0.29386 (17)	0.0364 (7)	
O5	0.5877 (3)	0.25815 (17)	0.25297 (17)	0.0417 (7)	
O6	0.7879 (3)	−0.24453 (18)	0.28088 (19)	0.0544 (9)	
H6B	0.8137	−0.2682	0.2396	0.082*	
O7	0.4396 (3)	0.2726 (2)	0.41757 (17)	0.0484 (8)	
O8	0.2440 (3)	0.34127 (19)	0.42558 (16)	0.0407 (7)	
O9	0.1528 (4)	0.7131 (2)	0.5514 (2)	0.0739 (11)	
H9A	0.2094	0.7309	0.5545	0.111*	
O10	0.1829 (3)	0.1677 (2)	0.0889 (2)	0.0597 (9)	
O11	0.3566 (3)	0.0745 (2)	0.10141 (17)	0.0491 (8)	
O12	0.1734 (3)	−0.2319 (2)	−0.0129 (2)	0.0671 (10)	
H12A	0.2349	−0.2718	−0.0334	0.081*	
O13	0.0898 (2)	0.27919 (17)	0.22449 (17)	0.0365 (7)	
O14	0.0091 (3)	0.17273 (18)	0.23625 (19)	0.0465 (8)	
O15	−0.1271 (3)	0.66678 (19)	0.14944 (19)	0.0634 (10)	
H15C	−0.1007	0.6757	0.1036	0.095*	
O16	0.3545 (2)	0.23006 (16)	0.18866 (15)	0.0299 (6)	
O17	0.4159 (3)	0.34714 (18)	0.16556 (15)	0.0364 (7)	
O18	0.3592 (3)	0.62591 (18)	−0.08311 (17)	0.0463 (8)	
H18B	0.4312	0.6262	−0.1059	0.069*	
C1	0.2257 (5)	0.0468 (3)	0.4865 (2)	0.0407 (11)	
C2	0.3512 (5)	−0.0014 (3)	0.4720 (2)	0.0477 (12)	
H2A	0.4122	0.0250	0.4453	0.057*	
C3	0.3889 (4)	−0.0878 (3)	0.4960 (3)	0.0466 (12)	
H3A	0.4740	−0.1187	0.4854	0.056*	
C4	0.2997 (4)	−0.1277 (3)	0.5358 (2)	0.0394 (11)	
C5	0.1735 (4)	−0.0816 (3)	0.5507 (3)	0.0410 (11)	
H5A	0.1128	−0.1085	0.5770	0.049*	
C6	0.1380 (4)	0.0046 (3)	0.5262 (2)	0.0418 (11)	
H6A	0.0528	0.0352	0.5366	0.050*	
C7	0.1858 (6)	0.1407 (3)	0.4592 (3)	0.0563 (14)	
H7A	0.2398	0.1711	0.4678	0.068*	
H7B	0.1003	0.1690	0.4887	0.068*	
C8	0.1921 (4)	0.1482 (3)	0.3758 (2)	0.0336 (10)	
C9	0.7528 (4)	0.0212 (3)	0.2546 (2)	0.0319 (9)	
C10	0.8073 (4)	−0.0319 (3)	0.1930 (2)	0.0376 (10)	
H10A	0.8363	−0.0077	0.1453	0.045*	
C11	0.8206 (4)	−0.1211 (3)	0.1997 (2)	0.0384 (10)	
H11A	0.8577	−0.1556	0.1569	0.046*	
C12	0.7789 (4)	−0.1581 (3)	0.2699 (3)	0.0370 (10)	
C13	0.7232 (4)	−0.1059 (3)	0.3331 (3)	0.0410 (11)	
H13A	0.6945	−0.1302	0.3807	0.049*	
C14	0.7105 (4)	−0.0176 (3)	0.3250 (2)	0.0382 (11)	
H14A	0.6727	0.0170	0.3678	0.046*	
C15	0.7384 (4)	0.1179 (2)	0.2472 (2)	0.0350 (10)	
H15A	0.7798	0.1313	0.2806	0.042*	
H15B	0.7823	0.1307	0.1955	0.042*	
C16	0.6061 (4)	0.1776 (3)	0.2652 (2)	0.0304 (9)	
C17	0.2678 (4)	0.4420 (3)	0.5429 (2)	0.0401 (11)	
C18	0.1482 (4)	0.4916 (3)	0.5393 (2)	0.0430 (11)	
H18A	0.0925	0.4640	0.5340	0.052*	
C19	0.1112 (5)	0.5826 (3)	0.5435 (2)	0.0471 (12)	
H19A	0.0301	0.6153	0.5427	0.057*	
C20	0.1936 (5)	0.6237 (3)	0.5488 (3)	0.0472 (12)	
C21	0.3114 (5)	0.5753 (3)	0.5539 (3)	0.0504 (12)	
H21A	0.3669	0.6031	0.5592	0.060*	
C22	0.3469 (4)	0.4858 (3)	0.5513 (3)	0.0458 (12)	
H22A	0.4265	0.4537	0.5552	0.055*	
C23	0.3104 (5)	0.3440 (3)	0.5360 (2)	0.0478 (12)	
H23A	0.2470	0.3202	0.5705	0.057*	
H23B	0.3875	0.3196	0.5509	0.057*	
C24	0.3328 (5)	0.3175 (3)	0.4558 (2)	0.0371 (10)	
C25	0.2749 (4)	−0.0063 (3)	−0.0148 (2)	0.0365 (10)	
C26	0.1592 (4)	−0.0134 (3)	0.0282 (2)	0.0423 (11)	
H26A	0.1021	0.0333	0.0576	0.051*	
C27	0.1263 (4)	−0.0887 (3)	0.0285 (3)	0.0467 (12)	
H27A	0.0480	−0.0923	0.0583	0.056*	
C28	0.2086 (4)	−0.1578 (3)	−0.0148 (3)	0.0427 (11)	
C29	0.3235 (4)	−0.1515 (3)	−0.0593 (3)	0.0462 (12)	
H29A	0.3792	−0.1979	−0.0897	0.055*	
C30	0.3563 (4)	−0.0773 (3)	−0.0591 (3)	0.0442 (11)	
H30A	0.4347	−0.0743	−0.0891	0.053*	
C31	0.3125 (4)	0.0758 (3)	−0.0168 (2)	0.0427 (11)	
H31A	0.4022	0.0633	−0.0407	0.051*	
H31B	0.2692	0.1206	−0.0474	0.051*	
C32	0.2810 (5)	0.1098 (3)	0.0617 (3)	0.0408 (11)	
C33	−0.1217 (4)	0.4080 (3)	0.1977 (3)	0.0387 (11)	
C34	−0.1014 (5)	0.4400 (3)	0.1243 (3)	0.0517 (13)	
H34A	−0.0866	0.4029	0.0854	0.062*	
C35	−0.1020 (5)	0.5256 (3)	0.1061 (3)	0.0497 (12)	
H35A	−0.0885	0.5456	0.0558	0.060*	
C36	−0.1226 (4)	0.5808 (3)	0.1627 (3)	0.0383 (10)	
C37	−0.1416 (5)	0.5502 (3)	0.2363 (3)	0.0485 (12)	
H37A	−0.1551	0.5874	0.2749	0.058*	
C38	−0.1408 (4)	0.4653 (3)	0.2536 (3)	0.0460 (12)	
H38A	−0.1533	0.4455	0.3040	0.055*	
C39	−0.1227 (4)	0.3157 (3)	0.2173 (3)	0.0514 (13)	
H39A	−0.1439	0.2944	0.1776	0.062*	
H39B	−0.1891	0.3156	0.2640	0.062*	
C40	−0.0020 (4)	0.2522 (3)	0.2276 (2)	0.0334 (10)	
C41	0.3569 (4)	0.3849 (3)	0.0245 (2)	0.0362 (10)	
C42	0.4591 (4)	0.3926 (3)	−0.0351 (2)	0.0415 (11)	
H42A	0.5275	0.3432	−0.0507	0.050*	
C43	0.4616 (4)	0.4719 (3)	−0.0717 (2)	0.0427 (11)	
H43A	0.5313	0.4757	−0.1114	0.051*	
C44	0.3614 (4)	0.5451 (3)	−0.0495 (2)	0.0358 (10)	
C45	0.2579 (4)	0.5391 (3)	0.0091 (2)	0.0401 (11)	
H45A	0.1898	0.5887	0.0245	0.048*	
C46	0.2562 (4)	0.4597 (3)	0.0446 (2)	0.0406 (11)	
H46A	0.1851	0.4559	0.0833	0.049*	
C47	0.3539 (5)	0.3003 (3)	0.0672 (2)	0.0426 (11)	
H47A	0.4172	0.2529	0.0365	0.051*	
H47B	0.2726	0.2922	0.0738	0.051*	
C48	0.3763 (4)	0.2935 (3)	0.1439 (2)	0.0303 (9)	
C49	0.2721 (4)	−0.0753 (3)	0.1783 (2)	0.0352 (10)	
H49A	0.3450	−0.0675	0.1441	0.042*	
C50	0.2544 (4)	−0.1574 (3)	0.1845 (3)	0.0394 (11)	
H50A	0.3150	−0.2029	0.1551	0.047*	
C51	0.1474 (4)	−0.1715 (3)	0.2343 (2)	0.0347 (10)	
C52	0.0606 (4)	−0.1001 (3)	0.2744 (3)	0.0417 (11)	
H52A	−0.0142	−0.1057	0.3080	0.050*	
C53	0.0841 (4)	−0.0208 (3)	0.2651 (3)	0.0395 (11)	
H53A	0.0233	0.0261	0.2925	0.047*	
C54	0.1774 (6)	−0.4055 (3)	0.2063 (4)	0.0668 (16)	
H54A	0.2194	−0.4465	0.1683	0.080*	
C55	0.1882 (5)	−0.3230 (3)	0.1905 (3)	0.0584 (14)	
H55A	0.2346	−0.3092	0.1427	0.070*	
C56	0.1301 (4)	−0.2600 (3)	0.2456 (3)	0.0415 (11)	
C57	0.0585 (5)	−0.2856 (3)	0.3136 (3)	0.0596 (15)	
H57A	0.0145	−0.2461	0.3526	0.072*	
C58	0.0528 (5)	−0.3702 (4)	0.3229 (4)	0.0755 (19)	
H58A	0.0035	−0.3856	0.3691	0.091*	
C59	0.5173 (4)	0.4507 (3)	0.2663 (3)	0.0375 (10)	
H59A	0.5819	0.4054	0.2389	0.045*	
C60	0.5361 (4)	0.5309 (3)	0.2618 (3)	0.0418 (11)	
H60A	0.6113	0.5390	0.2309	0.050*	
C61	0.4446 (4)	0.6000 (3)	0.3028 (2)	0.0378 (10)	
C62	0.3378 (5)	0.5814 (3)	0.3494 (3)	0.0424 (11)	
H62A	0.2751	0.6244	0.3806	0.051*	
C63	0.3235 (4)	0.4999 (3)	0.3500 (2)	0.0384 (10)	
H63A	0.2497	0.4901	0.3811	0.046*	
C64	0.3836 (7)	0.8402 (3)	0.3321 (3)	0.0690 (18)	
H64A	0.3290	0.8829	0.3666	0.083*	
C65	0.3752 (6)	0.7549 (3)	0.3472 (3)	0.0561 (14)	
H65A	0.3187	0.7415	0.3910	0.067*	
C66	0.4538 (5)	0.6908 (3)	0.2949 (3)	0.0443 (12)	
C67	0.5397 (5)	0.7153 (3)	0.2332 (3)	0.0598 (15)	
H67A	0.5959	0.6744	0.1974	0.072*	
C68	0.5411 (5)	0.8012 (3)	0.2252 (4)	0.0697 (17)	
H68A	0.6007	0.8160	0.1840	0.084*	
H2WA	0.161 (5)	0.4399 (13)	0.278 (3)	0.105*	
H1WA	0.458 (4)	0.063 (3)	0.258 (3)	0.105*	
H2WB	0.128 (5)	0.374 (3)	0.266 (3)	0.105*	
H1WB	0.442 (5)	−0.0102 (13)	0.246 (4)	0.105*	
H3WA	0.005 (4)	0.747 (4)	0.012 (3)	0.105*	
H3WB	−0.088 (5)	0.762 (3)	−0.020 (3)	0.105*	

### Atomic displacement parameters (Å^2^)

**Table:**

	*U*^11^	*U*^22^	*U*^33^	*U*^12^	*U*^13^	*U*^23^
Yb1	0.02696 (11)	0.01759 (9)	0.03884 (12)	−0.00890 (8)	−0.01179 (8)	0.00276 (8)
Yb2	0.02615 (10)	0.01619 (9)	0.03632 (11)	−0.00720 (7)	−0.01082 (8)	0.00041 (7)
N1	0.0283 (19)	0.0221 (17)	0.044 (2)	−0.0095 (15)	−0.0103 (16)	0.0004 (16)
N2	0.054 (3)	0.027 (2)	0.121 (4)	−0.016 (2)	−0.040 (3)	0.008 (3)
N3	0.039 (2)	0.0209 (17)	0.038 (2)	−0.0096 (16)	−0.0146 (17)	−0.0004 (15)
N4	0.076 (4)	0.037 (3)	0.109 (4)	−0.030 (3)	−0.062 (3)	0.024 (3)
O1W	0.0394 (18)	0.0174 (14)	0.0565 (19)	−0.0070 (13)	−0.0239 (15)	0.0002 (14)
O1	0.060 (2)	0.0504 (19)	0.0417 (18)	−0.0387 (17)	−0.0158 (16)	0.0049 (15)
O2W	0.0377 (18)	0.0230 (15)	0.059 (2)	−0.0068 (14)	−0.0229 (15)	−0.0001 (15)
O2	0.0312 (16)	0.0229 (14)	0.0399 (17)	−0.0130 (13)	−0.0079 (13)	0.0041 (12)
O3	0.059 (2)	0.0349 (18)	0.083 (3)	−0.0158 (17)	−0.036 (2)	0.0092 (17)
O3W	0.066 (3)	0.059 (2)	0.081 (3)	−0.025 (2)	−0.024 (2)	0.012 (2)
O4	0.0276 (16)	0.0203 (14)	0.063 (2)	−0.0072 (13)	−0.0140 (14)	−0.0062 (14)
O5	0.0309 (17)	0.0195 (15)	0.068 (2)	−0.0055 (13)	−0.0071 (15)	0.0023 (14)
O6	0.074 (2)	0.0241 (16)	0.067 (2)	−0.0135 (16)	−0.0241 (19)	−0.0009 (15)
O7	0.052 (2)	0.0413 (19)	0.0465 (19)	0.0009 (16)	−0.0190 (16)	−0.0062 (15)
O8	0.0415 (18)	0.0381 (17)	0.0443 (18)	−0.0136 (15)	−0.0110 (15)	−0.0059 (14)
O9	0.077 (3)	0.036 (2)	0.112 (3)	−0.0163 (19)	−0.030 (2)	−0.009 (2)
O10	0.057 (2)	0.057 (2)	0.064 (2)	−0.013 (2)	−0.0132 (19)	−0.0226 (19)
O11	0.064 (2)	0.056 (2)	0.0409 (18)	−0.0371 (18)	−0.0155 (17)	0.0042 (16)
O12	0.052 (2)	0.045 (2)	0.105 (3)	−0.0214 (18)	−0.008 (2)	−0.020 (2)
O13	0.0301 (16)	0.0225 (14)	0.064 (2)	−0.0068 (13)	−0.0235 (15)	−0.0046 (14)
O14	0.0354 (18)	0.0253 (16)	0.080 (2)	−0.0131 (14)	−0.0184 (16)	0.0114 (16)
O15	0.097 (3)	0.0290 (18)	0.061 (2)	−0.0235 (19)	−0.011 (2)	−0.0049 (16)
O16	0.0303 (15)	0.0222 (14)	0.0383 (16)	−0.0106 (12)	−0.0106 (13)	0.0057 (12)
O17	0.0482 (19)	0.0347 (16)	0.0374 (17)	−0.0281 (15)	−0.0140 (14)	0.0068 (13)
O18	0.050 (2)	0.0341 (17)	0.055 (2)	−0.0218 (15)	−0.0104 (16)	0.0105 (15)
C1	0.065 (3)	0.036 (3)	0.032 (2)	−0.026 (2)	−0.018 (2)	0.001 (2)
C2	0.066 (4)	0.058 (3)	0.035 (3)	−0.044 (3)	−0.011 (2)	0.003 (2)
C3	0.042 (3)	0.058 (3)	0.043 (3)	−0.017 (3)	−0.012 (2)	−0.007 (2)
C4	0.050 (3)	0.037 (3)	0.040 (3)	−0.019 (2)	−0.019 (2)	0.002 (2)
C5	0.043 (3)	0.036 (3)	0.051 (3)	−0.022 (2)	−0.017 (2)	0.007 (2)
C6	0.043 (3)	0.038 (3)	0.047 (3)	−0.013 (2)	−0.017 (2)	0.003 (2)
C7	0.100 (4)	0.038 (3)	0.041 (3)	−0.033 (3)	−0.024 (3)	0.006 (2)
C8	0.039 (3)	0.027 (2)	0.036 (2)	−0.014 (2)	−0.009 (2)	−0.0005 (19)
C9	0.025 (2)	0.030 (2)	0.041 (3)	−0.0033 (18)	−0.0133 (19)	−0.0027 (19)
C10	0.036 (3)	0.036 (2)	0.036 (2)	−0.005 (2)	−0.008 (2)	−0.002 (2)
C11	0.046 (3)	0.028 (2)	0.037 (3)	−0.005 (2)	−0.009 (2)	−0.010 (2)
C12	0.039 (3)	0.023 (2)	0.052 (3)	−0.0060 (19)	−0.019 (2)	−0.006 (2)
C13	0.046 (3)	0.032 (2)	0.042 (3)	−0.012 (2)	−0.008 (2)	0.003 (2)
C14	0.040 (3)	0.033 (2)	0.038 (3)	−0.004 (2)	−0.008 (2)	−0.013 (2)
C15	0.029 (2)	0.024 (2)	0.051 (3)	−0.0064 (18)	−0.010 (2)	−0.0049 (19)
C16	0.031 (2)	0.024 (2)	0.039 (2)	−0.0050 (18)	−0.0148 (19)	−0.0062 (18)
C17	0.051 (3)	0.039 (3)	0.029 (2)	−0.013 (2)	−0.009 (2)	−0.005 (2)
C18	0.047 (3)	0.045 (3)	0.040 (3)	−0.019 (2)	−0.009 (2)	−0.005 (2)
C19	0.046 (3)	0.042 (3)	0.051 (3)	−0.010 (2)	−0.012 (2)	−0.006 (2)
C20	0.058 (3)	0.037 (3)	0.044 (3)	−0.017 (3)	−0.006 (2)	−0.006 (2)
C21	0.054 (3)	0.047 (3)	0.053 (3)	−0.023 (3)	−0.008 (2)	−0.010 (2)
C22	0.040 (3)	0.045 (3)	0.051 (3)	−0.011 (2)	−0.011 (2)	−0.007 (2)
C23	0.065 (3)	0.040 (3)	0.035 (3)	−0.012 (2)	−0.012 (2)	0.000 (2)
C24	0.053 (3)	0.019 (2)	0.040 (3)	−0.014 (2)	−0.013 (2)	0.0044 (19)
C25	0.043 (3)	0.039 (3)	0.032 (2)	−0.015 (2)	−0.014 (2)	−0.001 (2)
C26	0.040 (3)	0.039 (3)	0.043 (3)	−0.008 (2)	−0.005 (2)	−0.012 (2)
C27	0.035 (3)	0.055 (3)	0.048 (3)	−0.020 (2)	0.001 (2)	−0.010 (2)
C28	0.038 (3)	0.038 (3)	0.055 (3)	−0.011 (2)	−0.015 (2)	−0.005 (2)
C29	0.041 (3)	0.033 (3)	0.059 (3)	−0.006 (2)	−0.007 (2)	−0.012 (2)
C30	0.033 (3)	0.046 (3)	0.049 (3)	−0.011 (2)	−0.002 (2)	−0.012 (2)
C31	0.054 (3)	0.039 (3)	0.036 (3)	−0.018 (2)	−0.010 (2)	−0.001 (2)
C32	0.052 (3)	0.038 (3)	0.042 (3)	−0.032 (2)	−0.009 (2)	0.003 (2)
C33	0.029 (2)	0.029 (2)	0.064 (3)	−0.0047 (19)	−0.027 (2)	0.002 (2)
C34	0.072 (4)	0.030 (3)	0.056 (3)	−0.003 (2)	−0.032 (3)	−0.012 (2)
C35	0.069 (3)	0.034 (3)	0.041 (3)	−0.008 (2)	−0.016 (2)	0.000 (2)
C36	0.041 (3)	0.023 (2)	0.048 (3)	−0.007 (2)	−0.011 (2)	0.000 (2)
C37	0.063 (3)	0.035 (3)	0.048 (3)	−0.014 (2)	−0.012 (2)	−0.012 (2)
C38	0.057 (3)	0.036 (3)	0.041 (3)	−0.011 (2)	−0.012 (2)	0.005 (2)
C39	0.038 (3)	0.027 (2)	0.094 (4)	−0.007 (2)	−0.030 (3)	0.005 (2)
C40	0.033 (2)	0.029 (2)	0.039 (3)	−0.0097 (19)	−0.012 (2)	0.0034 (19)
C41	0.050 (3)	0.034 (2)	0.038 (3)	−0.022 (2)	−0.025 (2)	0.009 (2)
C42	0.047 (3)	0.032 (2)	0.042 (3)	−0.008 (2)	−0.011 (2)	0.000 (2)
C43	0.044 (3)	0.044 (3)	0.039 (3)	−0.019 (2)	−0.005 (2)	0.006 (2)
C44	0.046 (3)	0.033 (2)	0.037 (2)	−0.020 (2)	−0.019 (2)	0.009 (2)
C45	0.041 (3)	0.035 (2)	0.041 (3)	−0.013 (2)	−0.008 (2)	0.007 (2)
C46	0.042 (3)	0.045 (3)	0.038 (3)	−0.023 (2)	−0.010 (2)	0.009 (2)
C47	0.065 (3)	0.036 (2)	0.040 (3)	−0.028 (2)	−0.024 (2)	0.009 (2)
C48	0.029 (2)	0.026 (2)	0.036 (2)	−0.0084 (18)	−0.0106 (18)	0.0064 (19)
C49	0.036 (2)	0.027 (2)	0.041 (3)	−0.012 (2)	−0.009 (2)	0.0024 (19)
C50	0.043 (3)	0.025 (2)	0.051 (3)	−0.007 (2)	−0.016 (2)	−0.003 (2)
C51	0.035 (2)	0.023 (2)	0.053 (3)	−0.0112 (19)	−0.021 (2)	0.004 (2)
C52	0.030 (2)	0.031 (2)	0.065 (3)	−0.014 (2)	−0.010 (2)	0.004 (2)
C53	0.033 (3)	0.026 (2)	0.055 (3)	−0.008 (2)	−0.006 (2)	−0.004 (2)
C54	0.094 (5)	0.037 (3)	0.083 (4)	−0.025 (3)	−0.037 (4)	−0.005 (3)
C55	0.096 (4)	0.031 (3)	0.060 (3)	−0.029 (3)	−0.029 (3)	0.001 (2)
C56	0.036 (3)	0.024 (2)	0.071 (3)	−0.008 (2)	−0.029 (2)	0.006 (2)
C57	0.045 (3)	0.030 (3)	0.088 (4)	−0.011 (2)	0.002 (3)	0.003 (3)
C58	0.048 (3)	0.041 (3)	0.124 (5)	−0.021 (3)	−0.010 (3)	0.030 (4)
C59	0.035 (3)	0.021 (2)	0.056 (3)	−0.0093 (19)	−0.012 (2)	−0.002 (2)
C60	0.037 (3)	0.034 (2)	0.061 (3)	−0.017 (2)	−0.015 (2)	0.002 (2)
C61	0.049 (3)	0.027 (2)	0.049 (3)	−0.017 (2)	−0.027 (2)	0.006 (2)
C62	0.056 (3)	0.026 (2)	0.046 (3)	−0.011 (2)	−0.017 (2)	0.000 (2)
C63	0.044 (3)	0.028 (2)	0.039 (3)	−0.010 (2)	−0.007 (2)	0.003 (2)
C64	0.124 (6)	0.030 (3)	0.077 (4)	−0.022 (3)	−0.064 (4)	−0.004 (3)
C65	0.106 (5)	0.029 (3)	0.047 (3)	−0.026 (3)	−0.037 (3)	0.001 (2)
C66	0.057 (3)	0.027 (2)	0.065 (3)	−0.018 (2)	−0.040 (3)	0.011 (2)
C67	0.045 (3)	0.029 (3)	0.110 (5)	−0.016 (2)	−0.028 (3)	0.012 (3)
C68	0.049 (3)	0.036 (3)	0.130 (5)	−0.018 (3)	−0.036 (4)	0.020 (3)

### Geometric parameters (Å, °)

**Table:**

Yb1—O1W	2.327 (3)	C17—C22	1.380 (6)
Yb1—O13	2.344 (3)	C17—C18	1.389 (6)
Yb1—O14	2.354 (3)	C17—C23	1.507 (6)
Yb1—O16	2.355 (3)	C18—C19	1.397 (6)
Yb1—O11	2.358 (3)	C18—H18A	0.9300
Yb1—O1	2.373 (3)	C19—C20	1.362 (6)
Yb1—N1	2.494 (3)	C19—H19A	0.9300
Yb1—O2	2.531 (3)	C20—C21	1.376 (6)
Yb1—O10	2.569 (3)	C21—C22	1.372 (6)
Yb1—C40	2.704 (4)	C21—H21A	0.9300
Yb1—C8	2.817 (4)	C22—H22A	0.9300
Yb1—C32	2.834 (4)	C23—C24	1.512 (6)
Yb2—O2	2.297 (3)	C23—H23A	0.9700
Yb2—O2W	2.338 (3)	C23—H23B	0.9700
Yb2—O5	2.340 (3)	C25—C26	1.378 (6)
Yb2—O4	2.364 (3)	C25—C30	1.392 (6)
Yb2—O8	2.379 (3)	C25—C31	1.517 (6)
Yb2—O7	2.470 (3)	C26—C27	1.386 (6)
Yb2—O16	2.488 (3)	C26—H26A	0.9300
Yb2—N3	2.509 (3)	C27—C28	1.367 (6)
Yb2—O17	2.522 (3)	C27—H27A	0.9300
Yb2—C16	2.728 (4)	C28—C29	1.373 (6)
Yb2—C24	2.806 (4)	C29—C30	1.370 (6)
Yb2—C48	2.885 (4)	C29—H29A	0.9300
N1—C49	1.333 (5)	C30—H30A	0.9300
N1—C53	1.336 (5)	C31—C32	1.508 (6)
N2—C54	1.312 (7)	C31—H31A	0.9700
N2—C58	1.317 (7)	C31—H31B	0.9700
N3—C59	1.342 (5)	C33—C34	1.369 (6)
N3—C63	1.343 (5)	C33—C38	1.387 (6)
N4—C64	1.309 (7)	C33—C39	1.493 (6)
N4—C68	1.334 (7)	C34—C35	1.380 (6)
O1W—H1WA	0.83 (5)	C34—H34A	0.9300
O1W—H1WB	0.835 (19)	C35—C36	1.367 (6)
O1—C8	1.237 (5)	C35—H35A	0.9300
O2W—H2WA	0.845 (19)	C36—C37	1.367 (6)
O2W—H2WB	0.84 (6)	C37—C38	1.368 (6)
O2—C8	1.268 (5)	C37—H37A	0.9300
O3—C4	1.379 (5)	C38—H38A	0.9300
O3—H3B	0.8200	C39—C40	1.508 (5)
O3W—H3WA	0.81 (6)	C39—H39A	0.9700
O3W—H3WB	0.82 (5)	C39—H39B	0.9700
O4—C16	1.268 (5)	C41—C46	1.383 (6)
O5—C16	1.252 (5)	C41—C42	1.385 (6)
O6—C12	1.360 (5)	C41—C47	1.499 (6)
O6—H6B	0.8200	C42—C43	1.380 (6)
O7—C24	1.262 (5)	C42—H42A	0.9300
O8—C24	1.258 (5)	C43—C44	1.369 (6)
O9—C20	1.368 (5)	C43—H43A	0.9300
O9—H9A	0.8200	C44—C45	1.378 (6)
O10—C32	1.232 (5)	C45—C46	1.373 (6)
O11—C32	1.270 (5)	C45—H45A	0.9300
O12—C28	1.376 (5)	C46—H46A	0.9300
O12—H12A	0.8200	C47—C48	1.501 (5)
O13—C40	1.264 (5)	C47—H47A	0.9700
O14—C40	1.242 (5)	C47—H47B	0.9700
O15—C36	1.367 (5)	C49—C50	1.390 (5)
O15—H15C	0.8200	C49—H49A	0.9300
O16—C48	1.279 (4)	C50—C51	1.377 (6)
O17—C48	1.253 (5)	C50—H50A	0.9300
O18—C44	1.373 (5)	C51—C52	1.381 (6)
O18—H18B	0.8200	C51—C56	1.488 (5)
C1—C2	1.383 (6)	C52—C53	1.372 (6)
C1—C6	1.386 (6)	C52—H52A	0.9300
C1—C7	1.511 (6)	C53—H53A	0.9300
C2—C3	1.384 (6)	C54—C55	1.365 (7)
C2—H2A	0.9300	C54—H54A	0.9300
C3—C4	1.376 (6)	C55—C56	1.383 (6)
C3—H3A	0.9300	C55—H55A	0.9300
C4—C5	1.381 (6)	C56—C57	1.381 (7)
C5—C6	1.382 (6)	C57—C58	1.375 (7)
C5—H5A	0.9300	C57—H57A	0.9300
C6—H6A	0.9300	C58—H58A	0.9300
C7—C8	1.511 (6)	C59—C60	1.370 (6)
C7—H7A	0.9700	C59—H59A	0.9300
C7—H7B	0.9700	C60—C61	1.381 (6)
C9—C10	1.368 (5)	C60—H60A	0.9300
C9—C14	1.394 (6)	C61—C62	1.383 (6)
C9—C15	1.510 (5)	C61—C66	1.489 (6)
C10—C11	1.393 (6)	C62—C63	1.375 (6)
C10—H10A	0.9300	C62—H62A	0.9300
C11—C12	1.375 (6)	C63—H63A	0.9300
C11—H11A	0.9300	C64—C65	1.399 (7)
C12—C13	1.384 (6)	C64—H64A	0.9300
C13—C14	1.379 (6)	C65—C66	1.393 (7)
C13—H13A	0.9300	C65—H65A	0.9300
C14—H14A	0.9300	C66—C67	1.385 (7)
C15—C16	1.499 (5)	C67—C68	1.380 (7)
C15—H15A	0.9700	C67—H67A	0.9300
C15—H15B	0.9700	C68—H68A	0.9300
			
O1W—Yb1—O13	146.19 (9)	C12—C11—C10	119.8 (4)
O1W—Yb1—O14	149.34 (11)	C12—C11—H11A	120.1
O13—Yb1—O14	55.00 (9)	C10—C11—H11A	120.1
O1W—Yb1—O16	79.69 (9)	O6—C12—C11	123.2 (4)
O13—Yb1—O16	73.79 (9)	O6—C12—C13	117.2 (4)
O14—Yb1—O16	128.36 (9)	C11—C12—C13	119.5 (4)
O1W—Yb1—O11	75.46 (11)	C14—C13—C12	119.7 (4)
O13—Yb1—O11	120.68 (11)	C14—C13—H13A	120.2
O14—Yb1—O11	115.67 (11)	C12—C13—H13A	120.2
O16—Yb1—O11	83.08 (10)	C13—C14—C9	121.8 (4)
O1W—Yb1—O1	78.50 (11)	C13—C14—H14A	119.1
O13—Yb1—O1	95.31 (11)	C9—C14—H14A	119.1
O14—Yb1—O1	77.02 (11)	C16—C15—C9	116.0 (3)
O16—Yb1—O1	117.33 (9)	C16—C15—H15A	108.3
O11—Yb1—O1	143.13 (11)	C9—C15—H15A	108.3
O1W—Yb1—N1	80.39 (10)	C16—C15—H15B	108.3
O13—Yb1—N1	130.72 (10)	C9—C15—H15B	108.3
O14—Yb1—N1	75.89 (10)	H15A—C15—H15B	107.4
O16—Yb1—N1	153.17 (10)	O5—C16—O4	118.8 (4)
O11—Yb1—N1	74.63 (11)	O5—C16—C15	119.1 (4)
O1—Yb1—N1	75.66 (10)	O4—C16—C15	122.1 (3)
O1W—Yb1—O2	74.23 (9)	O5—C16—Yb2	58.9 (2)
O13—Yb1—O2	75.80 (9)	O4—C16—Yb2	59.96 (19)
O14—Yb1—O2	104.26 (10)	C15—C16—Yb2	177.0 (3)
O16—Yb1—O2	65.22 (9)	C22—C17—C18	117.7 (4)
O11—Yb1—O2	139.24 (10)	C22—C17—C23	121.3 (4)
O1—Yb1—O2	52.44 (9)	C18—C17—C23	121.0 (4)
N1—Yb1—O2	125.34 (9)	C17—C18—C19	120.4 (4)
O1W—Yb1—O10	127.38 (11)	C17—C18—H18A	119.8
O13—Yb1—O10	74.34 (10)	C19—C18—H18A	119.8
O14—Yb1—O10	70.65 (11)	C20—C19—C18	120.2 (5)
O16—Yb1—O10	91.12 (10)	C20—C19—H19A	119.9
O11—Yb1—O10	51.96 (11)	C18—C19—H19A	119.9
O1—Yb1—O10	146.15 (11)	C19—C20—O9	117.6 (5)
N1—Yb1—O10	86.84 (11)	C19—C20—C21	119.9 (4)
O2—Yb1—O10	146.10 (10)	O9—C20—C21	122.5 (4)
O1W—Yb1—C40	164.73 (12)	C22—C21—C20	119.8 (4)
O13—Yb1—C40	27.85 (10)	C22—C21—H21A	120.1
O14—Yb1—C40	27.33 (11)	C20—C21—H21A	120.1
O16—Yb1—C40	101.07 (11)	C21—C22—C17	121.8 (5)
O11—Yb1—C40	119.81 (12)	C21—C22—H22A	119.1
O1—Yb1—C40	87.80 (12)	C17—C22—H22A	119.1
N1—Yb1—C40	102.88 (11)	C17—C23—C24	111.7 (3)
O2—Yb1—C40	92.12 (10)	C17—C23—H23A	109.3
O10—Yb1—C40	67.88 (12)	C24—C23—H23A	109.3
O1W—Yb1—C8	73.02 (11)	C17—C23—H23B	109.3
O13—Yb1—C8	86.96 (11)	C24—C23—H23B	109.3
O14—Yb1—C8	91.97 (12)	H23A—C23—H23B	107.9
O16—Yb1—C8	91.55 (10)	O8—C24—O7	118.7 (4)
O11—Yb1—C8	148.48 (12)	O8—C24—C23	119.8 (4)
O1—Yb1—C8	25.81 (10)	O7—C24—C23	121.5 (4)
N1—Yb1—C8	99.62 (11)	O8—C24—Yb2	57.5 (2)
O2—Yb1—C8	26.74 (10)	O7—C24—Yb2	61.6 (2)
O10—Yb1—C8	159.54 (12)	C23—C24—Yb2	172.3 (3)
C40—Yb1—C8	91.71 (12)	C26—C25—C30	117.3 (4)
O1W—Yb1—C32	101.77 (13)	C26—C25—C31	122.3 (4)
O13—Yb1—C32	98.37 (12)	C30—C25—C31	120.4 (4)
O14—Yb1—C32	91.95 (13)	C25—C26—C27	121.3 (4)
O16—Yb1—C32	88.96 (10)	C25—C26—H26A	119.3
O11—Yb1—C32	26.34 (12)	C27—C26—H26A	119.3
O1—Yb1—C32	152.95 (11)	C28—C27—C26	120.2 (4)
N1—Yb1—C32	77.72 (11)	C28—C27—H27A	119.9
O2—Yb1—C32	154.17 (10)	C26—C27—H27A	119.9
O10—Yb1—C32	25.78 (11)	C27—C28—C29	119.5 (4)
C40—Yb1—C32	93.49 (14)	C27—C28—O12	118.8 (4)
C8—Yb1—C32	174.57 (13)	C29—C28—O12	121.8 (4)
O2—Yb2—O2W	78.31 (10)	C30—C29—C28	120.3 (4)
O2—Yb2—O5	129.47 (9)	C30—C29—H29A	119.9
O2W—Yb2—O5	143.03 (11)	C28—C29—H29A	119.9
O2—Yb2—O4	75.27 (9)	C29—C30—C25	121.5 (4)
O2W—Yb2—O4	148.09 (9)	C29—C30—H30A	119.3
O5—Yb2—O4	54.92 (9)	C25—C30—H30A	119.3
O2—Yb2—O8	79.73 (9)	C32—C31—C25	111.9 (4)
O2W—Yb2—O8	75.22 (11)	C32—C31—H31A	109.2
O5—Yb2—O8	127.49 (10)	C25—C31—H31A	109.2
O4—Yb2—O8	116.60 (10)	C32—C31—H31B	109.2
O2—Yb2—O7	99.67 (10)	C25—C31—H31B	109.2
O2W—Yb2—O7	127.27 (11)	H31A—C31—H31B	107.9
O5—Yb2—O7	77.15 (11)	O10—C32—O11	119.9 (4)
O4—Yb2—O7	75.20 (10)	O10—C32—C31	121.9 (4)
O8—Yb2—O7	53.07 (10)	O11—C32—C31	118.1 (4)
O2—Yb2—O16	66.76 (9)	O10—C32—Yb1	65.0 (3)
O2W—Yb2—O16	77.51 (9)	O11—C32—Yb1	55.5 (2)
O5—Yb2—O16	91.24 (9)	C31—C32—Yb1	167.9 (3)
O4—Yb2—O16	75.83 (9)	C34—C33—C38	116.8 (4)
O8—Yb2—O16	140.25 (9)	C34—C33—C39	122.2 (4)
O7—Yb2—O16	150.36 (9)	C38—C33—C39	120.9 (4)
O2—Yb2—N3	153.08 (10)	C33—C34—C35	122.3 (4)
O2W—Yb2—N3	82.74 (10)	C33—C34—H34A	118.9
O5—Yb2—N3	76.38 (10)	C35—C34—H34A	118.9
O4—Yb2—N3	127.80 (10)	C36—C35—C34	119.5 (4)
O8—Yb2—N3	77.04 (10)	C36—C35—H35A	120.3
O7—Yb2—N3	76.91 (10)	C34—C35—H35A	120.3
O16—Yb2—N3	127.22 (9)	C35—C36—C37	119.4 (4)
O2—Yb2—O17	115.73 (9)	C35—C36—O15	123.0 (4)
O2W—Yb2—O17	73.04 (10)	C37—C36—O15	117.6 (4)
O5—Yb2—O17	72.48 (10)	C36—C37—C38	120.6 (4)
O4—Yb2—O17	102.88 (10)	C36—C37—H37A	119.7
O8—Yb2—O17	140.34 (10)	C38—C37—H37A	119.7
O7—Yb2—O17	143.07 (10)	C37—C38—C33	121.4 (4)
O16—Yb2—O17	51.59 (8)	C37—C38—H38A	119.3
N3—Yb2—O17	75.94 (9)	C33—C38—H38A	119.3
O2—Yb2—C16	102.67 (11)	C33—C39—C40	116.1 (4)
O2W—Yb2—C16	158.49 (11)	C33—C39—H39A	108.3
O5—Yb2—C16	27.25 (10)	C40—C39—H39A	108.3
O4—Yb2—C16	27.67 (10)	C33—C39—H39B	108.3
O8—Yb2—C16	126.26 (11)	C40—C39—H39B	108.3
O7—Yb2—C16	74.06 (11)	H39A—C39—H39B	107.4
O16—Yb2—C16	83.15 (10)	O14—C40—O13	119.9 (4)
N3—Yb2—C16	101.97 (11)	O14—C40—C39	120.0 (4)
O17—Yb2—C16	87.63 (11)	O13—C40—C39	120.1 (4)
O2—Yb2—C24	91.33 (11)	O14—C40—Yb1	60.4 (2)
O2W—Yb2—C24	100.83 (12)	O13—C40—Yb1	60.1 (2)
O5—Yb2—C24	102.01 (12)	C39—C40—Yb1	169.9 (3)
O4—Yb2—C24	97.39 (11)	C46—C41—C42	117.3 (4)
O8—Yb2—C24	26.48 (11)	C46—C41—C47	120.3 (4)
O7—Yb2—C24	26.71 (11)	C42—C41—C47	122.4 (4)
O16—Yb2—C24	157.99 (11)	C43—C42—C41	121.4 (4)
N3—Yb2—C24	73.57 (11)	C43—C42—H42A	119.3
O17—Yb2—C24	149.44 (10)	C41—C42—H42A	119.3
C16—Yb2—C24	100.63 (12)	C44—C43—C42	120.0 (4)
O2—Yb2—C48	90.60 (10)	C44—C43—H43A	120.0
O2W—Yb2—C48	70.62 (11)	C42—C43—H43A	120.0
O5—Yb2—C48	83.74 (11)	C43—C44—O18	122.6 (4)
O4—Yb2—C48	91.96 (10)	C43—C44—C45	119.9 (4)
O8—Yb2—C48	145.73 (11)	O18—C44—C45	117.5 (4)
O7—Yb2—C48	160.74 (11)	C46—C45—C44	119.6 (4)
O16—Yb2—C48	26.23 (9)	C46—C45—H45A	120.2
N3—Yb2—C48	101.00 (11)	C44—C45—H45A	120.2
O17—Yb2—C48	25.69 (9)	C45—C46—C41	121.9 (4)
C16—Yb2—C48	87.87 (11)	C45—C46—H46A	119.0
C24—Yb2—C48	170.64 (12)	C41—C46—H46A	119.0
C49—N1—C53	116.3 (4)	C41—C47—C48	115.1 (3)
C49—N1—Yb1	124.2 (3)	C41—C47—H47A	108.5
C53—N1—Yb1	119.4 (3)	C48—C47—H47A	108.5
C54—N2—C58	115.4 (5)	C41—C47—H47B	108.5
C59—N3—C63	115.8 (4)	C48—C47—H47B	108.5
C59—N3—Yb2	122.7 (3)	H47A—C47—H47B	107.5
C63—N3—Yb2	121.4 (3)	O17—C48—O16	118.9 (3)
C64—N4—C68	116.1 (5)	O17—C48—C47	122.7 (4)
Yb1—O1W—H1WA	122 (4)	O16—C48—C47	118.4 (3)
Yb1—O1W—H1WB	135 (4)	O17—C48—Yb2	60.7 (2)
H1WA—O1W—H1WB	103 (3)	O16—C48—Yb2	59.29 (19)
C8—O1—Yb1	97.5 (3)	C47—C48—Yb2	169.3 (3)
Yb2—O2W—H2WA	126 (4)	N1—C49—C50	123.2 (4)
Yb2—O2W—H2WB	130 (4)	N1—C49—H49A	118.4
H2WA—O2W—H2WB	101 (3)	C50—C49—H49A	118.4
C8—O2—Yb2	154.3 (3)	C51—C50—C49	120.1 (4)
C8—O2—Yb1	89.3 (2)	C51—C50—H50A	120.0
Yb2—O2—Yb1	114.25 (10)	C49—C50—H50A	120.0
C4—O3—H3B	109.5	C50—C51—C52	116.4 (4)
H3WA—O3W—H3WB	110 (3)	C50—C51—C56	121.1 (4)
C16—O4—Yb2	92.4 (2)	C52—C51—C56	122.5 (4)
C16—O5—Yb2	93.9 (2)	C53—C52—C51	120.3 (4)
C12—O6—H6B	109.5	C53—C52—H52A	119.8
C24—O7—Yb2	91.7 (2)	C51—C52—H52A	119.8
C24—O8—Yb2	96.1 (3)	N1—C53—C52	123.7 (4)
C20—O9—H9A	109.5	N1—C53—H53A	118.2
C32—O10—Yb1	89.2 (3)	C52—C53—H53A	118.2
C32—O11—Yb1	98.2 (3)	N2—C54—C55	124.7 (5)
C28—O12—H12A	109.5	N2—C54—H54A	117.7
C40—O13—Yb1	92.1 (2)	C55—C54—H54A	117.7
C40—O14—Yb1	92.2 (2)	C54—C55—C56	120.1 (5)
C36—O15—H15C	109.5	C54—C55—H55A	120.0
C48—O16—Yb1	141.9 (2)	C56—C55—H55A	120.0
C48—O16—Yb2	94.5 (2)	C57—C56—C55	115.7 (4)
Yb1—O16—Yb2	113.77 (10)	C57—C56—C51	121.7 (4)
C48—O17—Yb2	93.6 (2)	C55—C56—C51	122.6 (4)
C44—O18—H18B	109.5	C58—C57—C56	119.3 (5)
C2—C1—C6	117.0 (4)	C58—C57—H57A	120.4
C2—C1—C7	121.6 (4)	C56—C57—H57A	120.4
C6—C1—C7	121.4 (5)	N2—C58—C57	124.9 (6)
C1—C2—C3	122.1 (4)	N2—C58—H58A	117.6
C1—C2—H2A	119.0	C57—C58—H58A	117.6
C3—C2—H2A	119.0	N3—C59—C60	123.8 (4)
C4—C3—C2	119.6 (5)	N3—C59—H59A	118.1
C4—C3—H3A	120.2	C60—C59—H59A	118.1
C2—C3—H3A	120.2	C59—C60—C61	120.6 (4)
C3—C4—O3	122.6 (4)	C59—C60—H60A	119.7
C3—C4—C5	119.8 (4)	C61—C60—H60A	119.7
O3—C4—C5	117.6 (4)	C60—C61—C62	115.8 (4)
C4—C5—C6	119.6 (4)	C60—C61—C66	123.2 (4)
C4—C5—H5A	120.2	C62—C61—C66	120.8 (4)
C6—C5—H5A	120.2	C63—C62—C61	120.7 (4)
C5—C6—C1	121.8 (4)	C63—C62—H62A	119.7
C5—C6—H6A	119.1	C61—C62—H62A	119.7
C1—C6—H6A	119.1	N3—C63—C62	123.2 (4)
C1—C7—C8	112.3 (4)	N3—C63—H63A	118.4
C1—C7—H7A	109.1	C62—C63—H63A	118.4
C8—C7—H7A	109.1	N4—C64—C65	124.8 (6)
C1—C7—H7B	109.1	N4—C64—H64A	117.6
C8—C7—H7B	109.1	C65—C64—H64A	117.6
H7A—C7—H7B	107.9	C66—C65—C64	118.3 (5)
O1—C8—O2	120.2 (4)	C66—C65—H65A	120.9
O1—C8—C7	120.0 (4)	C64—C65—H65A	120.9
O2—C8—C7	119.8 (4)	C67—C66—C65	117.1 (4)
O1—C8—Yb1	56.7 (2)	C67—C66—C61	120.7 (4)
O2—C8—Yb1	64.0 (2)	C65—C66—C61	122.1 (5)
C7—C8—Yb1	171.5 (3)	C68—C67—C66	119.3 (5)
C10—C9—C14	117.3 (4)	C68—C67—H67A	120.4
C10—C9—C15	121.8 (4)	C66—C67—H67A	120.4
C14—C9—C15	120.9 (4)	N4—C68—C67	124.3 (6)
C9—C10—C11	121.9 (4)	N4—C68—H68A	117.9
C9—C10—H10A	119.1	C67—C68—H68A	117.9
C11—C10—H10A	119.1		
			
O1W—Yb1—N1—C49	−52.1 (3)	C14—C9—C10—C11	0.0 (6)
O13—Yb1—N1—C49	142.8 (3)	C15—C9—C10—C11	−180.0 (4)
O14—Yb1—N1—C49	147.5 (3)	C9—C10—C11—C12	−0.3 (7)
O16—Yb1—N1—C49	−9.6 (4)	C10—C11—C12—O6	179.6 (4)
O11—Yb1—N1—C49	25.3 (3)	C10—C11—C12—C13	0.4 (6)
O1—Yb1—N1—C49	−132.6 (3)	O6—C12—C13—C14	−179.4 (4)
O2—Yb1—N1—C49	−114.9 (3)	C11—C12—C13—C14	−0.2 (7)
O10—Yb1—N1—C49	76.6 (3)	C12—C13—C14—C9	−0.2 (7)
C40—Yb1—N1—C49	143.1 (3)	C10—C9—C14—C13	0.3 (6)
C8—Yb1—N1—C49	−122.9 (3)	C15—C9—C14—C13	−179.7 (4)
C32—Yb1—N1—C49	52.3 (3)	C10—C9—C15—C16	115.0 (4)
O1W—Yb1—N1—C53	123.5 (3)	C14—C9—C15—C16	−64.9 (5)
O13—Yb1—N1—C53	−41.6 (3)	Yb2—O5—C16—O4	1.2 (4)
O14—Yb1—N1—C53	−36.9 (3)	Yb2—O5—C16—C15	−177.4 (3)
O16—Yb1—N1—C53	166.0 (3)	Yb2—O4—C16—O5	−1.2 (4)
O11—Yb1—N1—C53	−159.1 (3)	Yb2—O4—C16—C15	177.4 (3)
O1—Yb1—N1—C53	43.0 (3)	C9—C15—C16—O5	−172.9 (4)
O2—Yb1—N1—C53	60.8 (3)	C9—C15—C16—O4	8.5 (6)
O10—Yb1—N1—C53	−107.7 (3)	O2—Yb2—C16—O5	170.6 (2)
C40—Yb1—N1—C53	−41.3 (3)	O2W—Yb2—C16—O5	80.4 (4)
C8—Yb1—N1—C53	52.7 (3)	O4—Yb2—C16—O5	178.7 (4)
C32—Yb1—N1—C53	−132.1 (3)	O8—Yb2—C16—O5	−103.0 (2)
O2—Yb2—N3—C59	−174.8 (3)	O7—Yb2—C16—O5	−92.8 (2)
O2W—Yb2—N3—C59	−129.4 (3)	O16—Yb2—C16—O5	106.3 (2)
O5—Yb2—N3—C59	19.9 (3)	N3—Yb2—C16—O5	−20.4 (3)
O4—Yb2—N3—C59	40.4 (3)	O17—Yb2—C16—O5	54.7 (2)
O8—Yb2—N3—C59	154.2 (3)	C24—Yb2—C16—O5	−95.6 (2)
O7—Yb2—N3—C59	99.6 (3)	C48—Yb2—C16—O5	80.4 (2)
O16—Yb2—N3—C59	−61.1 (3)	O2—Yb2—C16—O4	−8.2 (2)
O17—Yb2—N3—C59	−55.1 (3)	O2W—Yb2—C16—O4	−98.4 (4)
C16—Yb2—N3—C59	29.3 (3)	O5—Yb2—C16—O4	−178.7 (4)
C24—Yb2—N3—C59	127.0 (3)	O8—Yb2—C16—O4	78.3 (2)
C48—Yb2—N3—C59	−60.8 (3)	O7—Yb2—C16—O4	88.4 (2)
O2—Yb2—N3—C63	0.7 (4)	O16—Yb2—C16—O4	−72.4 (2)
O2W—Yb2—N3—C63	46.1 (3)	N3—Yb2—C16—O4	160.9 (2)
O5—Yb2—N3—C63	−164.6 (3)	O17—Yb2—C16—O4	−124.0 (2)
O4—Yb2—N3—C63	−144.1 (3)	C24—Yb2—C16—O4	85.6 (2)
O8—Yb2—N3—C63	−30.3 (3)	C48—Yb2—C16—O4	−98.3 (2)
O7—Yb2—N3—C63	−84.9 (3)	C22—C17—C18—C19	0.4 (6)
O16—Yb2—N3—C63	114.4 (3)	C23—C17—C18—C19	−178.1 (4)
O17—Yb2—N3—C63	120.4 (3)	C17—C18—C19—C20	2.1 (7)
C16—Yb2—N3—C63	−155.1 (3)	C18—C19—C20—O9	178.9 (4)
C24—Yb2—N3—C63	−57.4 (3)	C18—C19—C20—C21	−3.3 (7)
C48—Yb2—N3—C63	114.7 (3)	C19—C20—C21—C22	2.0 (7)
O1W—Yb1—O1—C8	74.7 (3)	O9—C20—C21—C22	179.8 (4)
O13—Yb1—O1—C8	−71.7 (3)	C20—C21—C22—C17	0.5 (7)
O14—Yb1—O1—C8	−123.9 (3)	C18—C17—C22—C21	−1.7 (7)
O16—Yb1—O1—C8	2.8 (3)	C23—C17—C22—C21	176.8 (4)
O11—Yb1—O1—C8	120.4 (3)	C22—C17—C23—C24	−107.1 (5)
N1—Yb1—O1—C8	157.6 (3)	C18—C17—C23—C24	71.4 (6)
O2—Yb1—O1—C8	−4.1 (2)	Yb2—O8—C24—O7	−7.4 (4)
O10—Yb1—O1—C8	−141.3 (3)	Yb2—O8—C24—C23	171.6 (3)
C40—Yb1—O1—C8	−98.5 (3)	Yb2—O7—C24—O8	7.1 (4)
C32—Yb1—O1—C8	168.1 (3)	Yb2—O7—C24—C23	−171.9 (4)
O2W—Yb2—O2—C8	−124.2 (6)	C17—C23—C24—O8	−59.9 (6)
O5—Yb2—O2—C8	83.4 (6)	C17—C23—C24—O7	119.1 (5)
O4—Yb2—O2—C8	73.9 (6)	O2—Yb2—C24—O8	−63.5 (2)
O8—Yb2—O2—C8	−47.3 (6)	O2W—Yb2—C24—O8	14.9 (2)
O7—Yb2—O2—C8	2.1 (6)	O5—Yb2—C24—O8	165.6 (2)
O16—Yb2—O2—C8	154.5 (6)	O4—Yb2—C24—O8	−138.8 (2)
N3—Yb2—O2—C8	−78.0 (6)	O7—Yb2—C24—O8	−172.6 (4)
O17—Yb2—O2—C8	171.3 (6)	O16—Yb2—C24—O8	−68.6 (4)
C16—Yb2—O2—C8	77.8 (6)	N3—Yb2—C24—O8	93.9 (2)
C24—Yb2—O2—C8	−23.4 (6)	O17—Yb2—C24—O8	89.8 (3)
C48—Yb2—O2—C8	165.7 (6)	C16—Yb2—C24—O8	−166.6 (2)
O2W—Yb2—O2—Yb1	81.21 (12)	O2—Yb2—C24—O7	109.1 (2)
O5—Yb2—O2—Yb1	−71.23 (15)	O2W—Yb2—C24—O7	−172.5 (2)
O4—Yb2—O2—Yb1	−80.71 (12)	O5—Yb2—C24—O7	−21.8 (3)
O8—Yb2—O2—Yb1	158.08 (13)	O4—Yb2—C24—O7	33.8 (3)
O7—Yb2—O2—Yb1	−152.49 (11)	O8—Yb2—C24—O7	172.6 (4)
O16—Yb2—O2—Yb1	−0.07 (9)	O16—Yb2—C24—O7	104.1 (4)
N3—Yb2—O2—Yb1	127.40 (19)	N3—Yb2—C24—O7	−93.5 (3)
O17—Yb2—O2—Yb1	16.71 (14)	O17—Yb2—C24—O7	−97.6 (3)
C16—Yb2—O2—Yb1	−76.80 (13)	C16—Yb2—C24—O7	6.0 (3)
C24—Yb2—O2—Yb1	−178.00 (13)	C30—C25—C26—C27	1.2 (7)
C48—Yb2—O2—Yb1	11.16 (12)	C31—C25—C26—C27	179.2 (4)
O1W—Yb1—O2—C8	−83.5 (2)	C25—C26—C27—C28	−0.5 (7)
O13—Yb1—O2—C8	112.4 (2)	C26—C27—C28—C29	−0.8 (7)
O14—Yb1—O2—C8	64.8 (2)	C26—C27—C28—O12	179.6 (4)
O16—Yb1—O2—C8	−169.2 (2)	C27—C28—C29—C30	1.4 (7)
O11—Yb1—O2—C8	−126.8 (2)	O12—C28—C29—C30	−179.0 (4)
O1—Yb1—O2—C8	4.0 (2)	C28—C29—C30—C25	−0.6 (7)
N1—Yb1—O2—C8	−17.8 (3)	C26—C25—C30—C29	−0.7 (7)
O10—Yb1—O2—C8	141.2 (2)	C31—C25—C30—C29	−178.7 (4)
C40—Yb1—O2—C8	89.6 (2)	C26—C25—C31—C32	46.3 (6)
C32—Yb1—O2—C8	−167.9 (3)	C30—C25—C31—C32	−135.8 (4)
O1W—Yb1—O2—Yb2	85.80 (12)	Yb1—O10—C32—O11	−8.4 (4)
O13—Yb1—O2—Yb2	−78.37 (12)	Yb1—O10—C32—C31	168.8 (4)
O14—Yb1—O2—Yb2	−125.93 (11)	Yb1—O11—C32—O10	9.3 (4)
O16—Yb1—O2—Yb2	0.08 (9)	Yb1—O11—C32—C31	−168.0 (3)
O11—Yb1—O2—Yb2	42.45 (19)	C25—C31—C32—O10	−95.8 (5)
O1—Yb1—O2—Yb2	173.27 (17)	C25—C31—C32—O11	81.5 (5)
N1—Yb1—O2—Yb2	151.42 (11)	C25—C31—C32—Yb1	26.5 (18)
O10—Yb1—O2—Yb2	−49.5 (2)	O1W—Yb1—C32—O10	−174.0 (2)
C40—Yb1—O2—Yb2	−101.14 (13)	O13—Yb1—C32—O10	−21.3 (3)
C8—Yb1—O2—Yb2	169.3 (3)	O14—Yb1—C32—O10	33.6 (3)
C32—Yb1—O2—Yb2	1.4 (3)	O16—Yb1—C32—O10	−94.7 (3)
O2—Yb2—O4—C16	171.8 (2)	O11—Yb1—C32—O10	−171.2 (4)
O2W—Yb2—O4—C16	136.7 (2)	O1—Yb1—C32—O10	98.3 (4)
O5—Yb2—O4—C16	0.7 (2)	N1—Yb1—C32—O10	108.7 (3)
O8—Yb2—O4—C16	−118.0 (2)	O2—Yb1—C32—O10	−95.9 (4)
O7—Yb2—O4—C16	−83.8 (2)	C40—Yb1—C32—O10	6.3 (3)
O16—Yb2—O4—C16	102.5 (2)	O1W—Yb1—C32—O11	−2.8 (3)
N3—Yb2—O4—C16	−23.9 (3)	O13—Yb1—C32—O11	149.9 (2)
O17—Yb2—O4—C16	58.2 (2)	O14—Yb1—C32—O11	−155.2 (2)
C24—Yb2—O4—C16	−98.8 (2)	O16—Yb1—C32—O11	76.4 (2)
C48—Yb2—O4—C16	81.6 (2)	O1—Yb1—C32—O11	−90.5 (3)
O2—Yb2—O5—C16	−11.9 (3)	N1—Yb1—C32—O11	−80.1 (2)
O2W—Yb2—O5—C16	−143.1 (2)	O2—Yb1—C32—O11	75.2 (4)
O4—Yb2—O5—C16	−0.7 (2)	O10—Yb1—C32—O11	171.2 (4)
O8—Yb2—O5—C16	98.0 (2)	C40—Yb1—C32—O11	177.4 (2)
O7—Yb2—O5—C16	80.1 (2)	O1W—Yb1—C32—C31	58.4 (16)
O16—Yb2—O5—C16	−72.4 (2)	O13—Yb1—C32—C31	−148.9 (16)
N3—Yb2—O5—C16	159.5 (3)	O14—Yb1—C32—C31	−94.0 (16)
O17—Yb2—O5—C16	−121.2 (2)	O16—Yb1—C32—C31	137.6 (16)
C24—Yb2—O5—C16	89.9 (2)	O11—Yb1—C32—C31	61.2 (16)
C48—Yb2—O5—C16	−97.6 (2)	O1—Yb1—C32—C31	−29.3 (18)
O2—Yb2—O7—C24	−73.4 (2)	N1—Yb1—C32—C31	−18.9 (16)
O2W—Yb2—O7—C24	9.3 (3)	O2—Yb1—C32—C31	136.5 (15)
O5—Yb2—O7—C24	158.1 (3)	O10—Yb1—C32—C31	−127.6 (17)
O4—Yb2—O7—C24	−145.2 (3)	C40—Yb1—C32—C31	−121.3 (16)
O8—Yb2—O7—C24	−4.1 (2)	C38—C33—C34—C35	−1.2 (7)
O16—Yb2—O7—C24	−132.7 (2)	C39—C33—C34—C35	179.2 (4)
N3—Yb2—O7—C24	79.4 (2)	C33—C34—C35—C36	0.5 (8)
O17—Yb2—O7—C24	123.0 (3)	C34—C35—C36—C37	0.4 (7)
C16—Yb2—O7—C24	−173.9 (3)	C34—C35—C36—O15	−178.6 (4)
C48—Yb2—O7—C24	165.3 (3)	C35—C36—C37—C38	−0.4 (7)
O2—Yb2—O8—C24	114.6 (2)	O15—C36—C37—C38	178.7 (4)
O2W—Yb2—O8—C24	−164.9 (3)	C36—C37—C38—C33	−0.4 (7)
O5—Yb2—O8—C24	−17.9 (3)	C34—C33—C38—C37	1.2 (7)
O4—Yb2—O8—C24	46.9 (3)	C39—C33—C38—C37	−179.2 (4)
O7—Yb2—O8—C24	4.1 (2)	C34—C33—C39—C40	97.4 (5)
O16—Yb2—O8—C24	146.9 (2)	C38—C33—C39—C40	−82.1 (6)
N3—Yb2—O8—C24	−79.1 (2)	Yb1—O14—C40—O13	−8.8 (4)
O17—Yb2—O8—C24	−127.2 (2)	Yb1—O14—C40—C39	168.4 (4)
C16—Yb2—O8—C24	16.4 (3)	Yb1—O13—C40—O14	8.9 (4)
C48—Yb2—O8—C24	−169.7 (2)	Yb1—O13—C40—C39	−168.3 (4)
O1W—Yb1—O10—C32	7.4 (3)	C33—C39—C40—O14	−172.5 (4)
O13—Yb1—O10—C32	158.1 (3)	C33—C39—C40—O13	4.6 (7)
O14—Yb1—O10—C32	−144.1 (3)	C33—C39—C40—Yb1	−83.2 (17)
O16—Yb1—O10—C32	85.3 (3)	O1W—Yb1—C40—O14	−91.5 (5)
O11—Yb1—O10—C32	5.0 (2)	O13—Yb1—C40—O14	−171.2 (4)
O1—Yb1—O10—C32	−126.1 (3)	O16—Yb1—C40—O14	177.1 (2)
N1—Yb1—O10—C32	−67.9 (3)	O11—Yb1—C40—O14	88.8 (3)
O2—Yb1—O10—C32	129.0 (3)	O1—Yb1—C40—O14	−65.5 (3)
C40—Yb1—O10—C32	−173.2 (3)	N1—Yb1—C40—O14	9.3 (3)
C8—Yb1—O10—C32	−177.3 (3)	O2—Yb1—C40—O14	−117.7 (3)
O1W—Yb1—O11—C32	177.1 (3)	O10—Yb1—C40—O14	90.4 (3)
O13—Yb1—O11—C32	−35.3 (3)	C8—Yb1—C40—O14	−91.0 (3)
O14—Yb1—O11—C32	27.7 (3)	C32—Yb1—C40—O14	87.5 (3)
O16—Yb1—O11—C32	−101.8 (2)	O1W—Yb1—C40—O13	79.7 (5)
O1—Yb1—O11—C32	130.7 (2)	O14—Yb1—C40—O13	171.2 (4)
N1—Yb1—O11—C32	93.3 (2)	O16—Yb1—C40—O13	−11.7 (2)
O2—Yb1—O11—C32	−139.8 (2)	O11—Yb1—C40—O13	−100.0 (2)
O10—Yb1—O11—C32	−4.9 (2)	O1—Yb1—C40—O13	105.7 (2)
C40—Yb1—O11—C32	−2.9 (3)	N1—Yb1—C40—O13	−179.5 (2)
C8—Yb1—O11—C32	176.6 (2)	O2—Yb1—C40—O13	53.4 (2)
O1W—Yb1—O13—C40	−152.3 (2)	O10—Yb1—C40—O13	−98.4 (3)
O14—Yb1—O13—C40	−4.9 (2)	C8—Yb1—C40—O13	80.2 (2)
O16—Yb1—O13—C40	168.0 (3)	C32—Yb1—C40—O13	−101.3 (2)
O11—Yb1—O13—C40	96.6 (3)	O1W—Yb1—C40—C39	173.3 (14)
O1—Yb1—O13—C40	−75.0 (2)	O13—Yb1—C40—C39	93.7 (17)
N1—Yb1—O13—C40	0.6 (3)	O14—Yb1—C40—C39	−95.2 (17)
O2—Yb1—O13—C40	−124.1 (2)	O16—Yb1—C40—C39	81.9 (17)
O10—Yb1—O13—C40	72.1 (2)	O11—Yb1—C40—C39	−6.4 (17)
C8—Yb1—O13—C40	−99.5 (2)	O1—Yb1—C40—C39	−160.6 (17)
C32—Yb1—O13—C40	81.6 (3)	N1—Yb1—C40—C39	−85.9 (17)
O1W—Yb1—O14—C40	148.9 (2)	O2—Yb1—C40—C39	147.1 (17)
O13—Yb1—O14—C40	5.0 (2)	O10—Yb1—C40—C39	−4.7 (16)
O16—Yb1—O14—C40	−3.6 (3)	C8—Yb1—C40—C39	173.9 (17)
O11—Yb1—O14—C40	−105.7 (3)	C32—Yb1—C40—C39	−7.7 (17)
O1—Yb1—O14—C40	111.1 (3)	C46—C41—C42—C43	1.5 (6)
N1—Yb1—O14—C40	−170.7 (3)	C47—C41—C42—C43	−177.6 (4)
O2—Yb1—O14—C40	65.9 (3)	C41—C42—C43—C44	−0.3 (7)
O10—Yb1—O14—C40	−79.1 (3)	C42—C43—C44—O18	179.1 (4)
C8—Yb1—O14—C40	89.9 (3)	C42—C43—C44—C45	−0.4 (6)
C32—Yb1—O14—C40	−93.8 (3)	C43—C44—C45—C46	−0.2 (6)
O1W—Yb1—O16—C48	148.2 (4)	O18—C44—C45—C46	−179.8 (4)
O13—Yb1—O16—C48	−53.0 (4)	C44—C45—C46—C41	1.5 (6)
O14—Yb1—O16—C48	−45.6 (4)	C42—C41—C46—C45	−2.2 (6)
O11—Yb1—O16—C48	71.8 (4)	C47—C41—C46—C45	177.0 (4)
O1—Yb1—O16—C48	−140.6 (4)	C46—C41—C47—C48	−76.1 (5)
N1—Yb1—O16—C48	105.6 (4)	C42—C41—C47—C48	103.0 (5)
O2—Yb1—O16—C48	−134.5 (4)	Yb2—O17—C48—O16	−12.1 (4)
O10—Yb1—O16—C48	20.4 (4)	Yb2—O17—C48—C47	168.2 (4)
C40—Yb1—O16—C48	−47.3 (4)	Yb1—O16—C48—O17	151.3 (3)
C8—Yb1—O16—C48	−139.4 (4)	Yb2—O16—C48—O17	12.2 (4)
C32—Yb1—O16—C48	46.0 (4)	Yb1—O16—C48—C47	−28.9 (6)
O1W—Yb1—O16—Yb2	−77.35 (12)	Yb2—O16—C48—C47	−168.0 (3)
O13—Yb1—O16—Yb2	81.46 (11)	Yb1—O16—C48—Yb2	139.1 (4)
O14—Yb1—O16—Yb2	88.82 (14)	C41—C47—C48—O17	−11.8 (6)
O11—Yb1—O16—Yb2	−153.75 (13)	C41—C47—C48—O16	168.4 (4)
O1—Yb1—O16—Yb2	−6.14 (15)	C41—C47—C48—Yb2	93.7 (15)
N1—Yb1—O16—Yb2	−120.0 (2)	O2—Yb2—C48—O17	168.4 (2)
O2—Yb1—O16—Yb2	−0.07 (8)	O2W—Yb2—C48—O17	91.0 (2)
O10—Yb1—O16—Yb2	154.80 (12)	O5—Yb2—C48—O17	−61.9 (2)
C40—Yb1—O16—Yb2	87.13 (13)	O4—Yb2—C48—O17	−116.3 (2)
C8—Yb1—O16—Yb2	−4.92 (12)	O8—Yb2—C48—O17	95.9 (3)
C32—Yb1—O16—Yb2	−179.51 (14)	O7—Yb2—C48—O17	−68.9 (4)
O2—Yb2—O16—C48	153.9 (2)	O16—Yb2—C48—O17	−167.7 (4)
O2W—Yb2—O16—C48	71.4 (2)	N3—Yb2—C48—O17	12.8 (2)
O5—Yb2—O16—C48	−73.1 (2)	C16—Yb2—C48—O17	−88.9 (2)
O4—Yb2—O16—C48	−126.3 (2)	O2—Yb2—C48—O16	−23.9 (2)
O8—Yb2—O16—C48	118.9 (2)	O2W—Yb2—C48—O16	−101.3 (2)
O7—Yb2—O16—C48	−138.8 (3)	O5—Yb2—C48—O16	105.8 (2)
N3—Yb2—O16—C48	0.7 (3)	O4—Yb2—C48—O16	51.4 (2)
O17—Yb2—O16—C48	−6.8 (2)	O8—Yb2—C48—O16	−96.4 (3)
C16—Yb2—O16—C48	−99.2 (2)	O7—Yb2—C48—O16	98.8 (4)
C24—Yb2—O16—C48	159.4 (3)	N3—Yb2—C48—O16	−179.4 (2)
O2—Yb2—O16—Yb1	0.08 (9)	O17—Yb2—C48—O16	167.7 (4)
O2W—Yb2—O16—Yb1	−82.41 (12)	C16—Yb2—C48—O16	78.8 (2)
O5—Yb2—O16—Yb1	133.13 (11)	O2—Yb2—C48—C47	56.8 (15)
O4—Yb2—O16—Yb1	79.89 (11)	O2W—Yb2—C48—C47	−20.6 (15)
O8—Yb2—O16—Yb1	−34.86 (19)	O5—Yb2—C48—C47	−173.5 (15)
O7—Yb2—O16—Yb1	67.4 (2)	O4—Yb2—C48—C47	132.1 (15)
N3—Yb2—O16—Yb1	−153.10 (11)	O8—Yb2—C48—C47	−15.7 (16)
O17—Yb2—O16—Yb1	−160.54 (16)	O7—Yb2—C48—C47	179.5 (13)
C16—Yb2—O16—Yb1	107.05 (13)	O16—Yb2—C48—C47	80.7 (15)
C48—Yb2—O16—Yb1	−153.8 (3)	N3—Yb2—C48—C47	−98.8 (15)
O2—Yb2—O17—C48	−12.9 (3)	O17—Yb2—C48—C47	−111.6 (15)
O2W—Yb2—O17—C48	−80.4 (2)	C16—Yb2—C48—C47	159.5 (15)
O5—Yb2—O17—C48	113.1 (2)	C53—N1—C49—C50	−2.3 (6)
O4—Yb2—O17—C48	66.8 (2)	Yb1—N1—C49—C50	173.5 (3)
O8—Yb2—O17—C48	−118.6 (2)	N1—C49—C50—C51	0.2 (6)
O7—Yb2—O17—C48	149.2 (2)	C49—C50—C51—C52	1.7 (6)
O16—Yb2—O17—C48	6.9 (2)	C49—C50—C51—C56	−176.1 (4)
N3—Yb2—O17—C48	−167.0 (2)	C50—C51—C52—C53	−1.4 (6)
C16—Yb2—O17—C48	90.0 (2)	C56—C51—C52—C53	176.3 (4)
C6—C1—C2—C3	−0.4 (6)	C49—N1—C53—C52	2.6 (6)
C7—C1—C2—C3	−179.3 (4)	Yb1—N1—C53—C52	−173.4 (3)
C1—C2—C3—C4	−0.1 (7)	C51—C52—C53—N1	−0.8 (7)
C2—C3—C4—O3	−179.9 (4)	C58—N2—C54—C55	1.1 (8)
C2—C3—C4—C5	0.7 (6)	N2—C54—C55—C56	1.5 (9)
C3—C4—C5—C6	−0.8 (6)	C54—C55—C56—C57	−3.0 (7)
O3—C4—C5—C6	179.8 (4)	C54—C55—C56—C51	174.5 (4)
C4—C5—C6—C1	0.3 (7)	C50—C51—C56—C57	151.4 (4)
C2—C1—C6—C5	0.3 (6)	C52—C51—C56—C57	−26.2 (6)
C7—C1—C6—C5	179.3 (4)	C50—C51—C56—C55	−25.9 (6)
C2—C1—C7—C8	78.5 (5)	C52—C51—C56—C55	156.5 (5)
C6—C1—C7—C8	−100.4 (5)	C55—C56—C57—C58	2.1 (7)
Yb1—O1—C8—O2	7.6 (4)	C51—C56—C57—C58	−175.4 (4)
Yb1—O1—C8—C7	−170.9 (4)	C54—N2—C58—C57	−2.0 (9)
Yb2—O2—C8—O1	−164.0 (4)	C56—C57—C58—N2	0.4 (9)
Yb1—O2—C8—O1	−7.1 (4)	C63—N3—C59—C60	−3.8 (6)
Yb2—O2—C8—C7	14.5 (9)	Yb2—N3—C59—C60	171.9 (3)
Yb1—O2—C8—C7	171.4 (4)	N3—C59—C60—C61	1.6 (7)
Yb2—O2—C8—Yb1	−157.0 (6)	C59—C60—C61—C62	2.3 (6)
C1—C7—C8—O2	−132.3 (4)	C59—C60—C61—C66	−173.9 (4)
O1W—Yb1—C8—O1	−98.8 (3)	C60—C61—C62—C63	−3.7 (6)
O13—Yb1—C8—O1	108.8 (3)	C66—C61—C62—C63	172.5 (4)
O14—Yb1—C8—O1	54.0 (3)	C59—N3—C63—C62	2.3 (6)
O16—Yb1—C8—O1	−177.5 (3)	Yb2—N3—C63—C62	−173.6 (3)
O11—Yb1—C8—O1	−98.2 (3)	C61—C62—C63—N3	1.5 (7)
N1—Yb1—C8—O1	−22.0 (3)	C68—N4—C64—C65	−1.3 (8)
O2—Yb1—C8—O1	172.7 (4)	N4—C64—C65—C66	−1.8 (8)
O10—Yb1—C8—O1	85.1 (4)	C64—C65—C66—C67	3.3 (7)
C40—Yb1—C8—O1	81.4 (3)	C64—C65—C66—C61	−174.9 (4)
O1W—Yb1—C8—O2	88.5 (2)	C60—C61—C66—C67	15.8 (6)
O13—Yb1—C8—O2	−63.9 (2)	C62—C61—C66—C67	−160.2 (4)
O14—Yb1—C8—O2	−118.7 (2)	C60—C61—C66—C65	−166.1 (4)
O16—Yb1—C8—O2	9.8 (2)	C62—C61—C66—C65	17.9 (6)
O11—Yb1—C8—O2	89.1 (3)	C65—C66—C67—C68	−1.9 (7)
O1—Yb1—C8—O2	−172.7 (4)	C61—C66—C67—C68	176.4 (4)
N1—Yb1—C8—O2	165.3 (2)	C64—N4—C68—C67	2.9 (8)
O10—Yb1—C8—O2	−87.6 (4)	C66—C67—C68—N4	−1.3 (8)
C40—Yb1—C8—O2	−91.3 (2)		

### Hydrogen-bond geometry (Å, °)

**Table:**

*D*—H···*A*	*D*—H	H···*A*	*D*···*A*	*D*—H···*A*
O6—H6B···O15^i^	0.82	1.90	2.715 (4)	174
O9—H9A···O3^ii^	0.82	1.94	2.761 (5)	173
O12—H12A···O18^iii^	0.82	1.92	2.735 (5)	171
O15—H15C···O3W	0.82	1.85	2.645 (5)	162
O2W—H2WA···N2^ii^	0.85 (2)	1.98 (2)	2.820 (5)	176 (6)
O1W—H1WA···O4	0.83 (5)	1.95 (3)	2.731 (4)	156 (5)
O2W—H2WB···O13	0.84 (6)	2.02 (3)	2.741 (4)	144 (5)
O1W—H1WB···N4^iii^	0.84 (2)	1.98 (3)	2.779 (5)	159 (6)
O3W—H3WA···O12^ii^	0.81 (6)	2.01 (3)	2.797 (5)	164 (7)

Symmetry codes: (i) *x*+1, *y*−1, *z*; (ii) *x*, *y*+1, *z*; (iii) *x*, *y*−1, *z*.
